# Nondestructive classification of soft rot disease in napa cabbage using hyperspectral imaging analysis

**DOI:** 10.1038/s41598-022-19169-6

**Published:** 2022-08-29

**Authors:** Hyeyeon Song, So-Ra Yoon, Yun-Mi Dang, Ji-Su Yang, In Min Hwang, Ji-Hyoung Ha

**Affiliations:** Hygienic Safety and Distribution Research Group, World Institute of Kimchi, 86 Kimchi-ro, Nam-gu, Gwangju, 61755 Republic of Korea

**Keywords:** Microbiology, Plant sciences

## Abstract

Identification of soft rot disease in napa cabbage, an essential ingredient of kimchi, is challenging at the industrial scale. Therefore, nondestructive imaging techniques are necessary. Here, we investigated the potential of hyperspectral imaging (HSI) processing in the near-infrared region (900–1700 nm) for classifying napa cabbage quality using nondestructive measurements. We determined the microbiological and physicochemical qualitative properties of napa cabbage for intercomparison of HSI information, extracted HSI characteristics from hyperspectral images to predict and classify freshness, and established a novel approach for classifying healthy and rotten napa cabbage. The second derivative Savitzky–Golay method for data preprocessing was implemented, followed by wavelength selection using variable importance in projection scores. For multivariate data of the classification models, partial least square discriminant analysis (PLS-DA), support vector machine (SVM), and random forests were used for predicting cabbage conditions. The SVM model accurately distinguished the cabbage exhibiting soft rot disease symptoms from the healthy cabbage. This study presents the potential of HSI systems for separating soft rot disease-infected napa cabbages from healthy napa cabbages using the SVM model, especially under the most effective wavelengths (970, 980, 1180, 1070, 1120, and 978 nm), prior to processing. These results are applicable to industrial multispectral images.

## Introduction

Pectinolytic enterobacteria of the genus *Pectobacterium* (e.g., *Pectobacterium carotovorum* subsp. *carotovorum*, PCC) are the most common cause of major diseases, such as soft rot disease, in healthy vegetables, causing serious deterioration in quality of various healthy produce^1^. Soft rot is an important attribute that significantly influences cabbage texture through enzymatic maceration^2^. In addition, sticky patches, a common symptom of spoilage, are the root cause of unpleasant odors and may adversely affect adjacent products. Therefore, research has been performed to identify plant pathogens associated with soft rot disease and to develop protocols for detection and classification of infected vegetables^3,4^. In addition, PCC has long-lasting biological properties in environmental matrices, such as surface water, groundwater, and soil, and infected agricultural products, such as seeds and vegetables^5^. Recently, Kang et al. reported that PCC contamination seriously affected the quality of kimchi due to the deterioration of commercially cut healthy napa cabbage (*Brassica rapa* L. subsp. *pekinensis* Hanelt)^6^.

Napa cabbage is an essential ingredient of kimchi, a traditional Korean fermented food standardized by the Codex Alimentarius Commission (CXS 223-2001) in 2001^7^. It accounts for 70‒80% of kimchi ingredients, thus directly affecting the quality of kimchi^8^. To improve kimchi quality, identifying infected napa cabbage before its processing is crucial. Generally, during the kimchi manufacturing process, soft rot disease of cabbage can be clearly determined while halving the cabbage; however, identification of soft rot disease symptoms in napa cabbage by the naked eye is challenging because of the large amount of cabbage that passes through the cutting machine. Moreover, even if an experienced employee visually inspects the softness of the cabbage tissue, efficiency issues may arise because of the unclear criteria of the operator. Therefore, developing rapid, nondestructive, and unbiased detection methods based on imaging analysis techniques for maintaining kimchi quality is necessary.

Research related to monitoring technologies for detecting soft rot symptoms has been conducted. Numerous studies have reported that several plant pathogens (e.g., *Botrytis cinerea*, *Fusarium sambucinum*, *Pectobacterium carotovorum* ssp. *atrosepticum*, *P. carotovorum* ssp. *carotovorum*, *Phytophthora infestans*, and *Pythium ultimum*) produce volatile markers that can be used to identify fruit and vegetable infections. Morath et al. reported that volatile compound (VC) emanation by fungi has biotechnological potential in the control of postharvest decay^9^. Strobel demonstrated the application of VCs for controlling postharvest fruit diseases using *Muscodor albus*^10^. Extensive evidence suggests that the VCs generated by antagonist bacteria could be effective in controlling postharvest decay caused by plant pathogens; however, the related studies often remain unreported^11,12^. Several studies have demonstrated that plant pathogenic bacteria can alter the profiles of VCs emitted from healthy vegetable tissues^13,14^. Interestingly, these biological characteristics can be considered specific disease markers. Several monitoring studies on VC emission using nondestructive methods have been conducted; however, few studies have monitored and classified the pathogenic symptoms of PCC-infected postharvest cabbage using imaging analysis techniques based on an active sensing system.

Hyperspectral imaging (HSI) is an innovative platform technique that can integrate spectroscopy^15^ and computer vision^16^ and simultaneously provide information on the spatial and spectral properties of samples. It has been widely employed in the evaluation of food safety and quality^17,18^, to study defects or bruise classification^19,20^, firmness, and soluble solid content^21^, and to monitor pear quality^22^. As a nondestructive technology, HSI has also been applied for early detection of fruit diseases in apples and peaches^16,20^. Although several lab-scale studies based on HSI have been performed to classify the spectral characteristics of bean, potato, tomato, and lettuce diseases^23,24^, no study has focused on soft rot in napa cabbage. Hyperspectral image data are formatted as three-dimensional hypercubes with two spatial images and one spectral wavelength. Because each individual pixel in a hyperspectral image has spectrum information about a specific position in a sample, various multivariate statistical methods, such as principal component analysis (PCA), partial least squares (PLS), linear discriminant analysis (LDA), and Fishers discriminant analysis (FDA) can be used to classify specific samples. Among them, the PLS method is the most commonly employed chemometric approach for multivariate data analysis. Furthermore, PLS in classification models is a practical and powerful method based on binary classification of highly correlated variables^25^. The partial least square discriminant analysis (PLS-DA) model has been previously evaluated in similar studies and has been recognized and recommended for its high accuracy (ACC) and simplicity^26,27^. The random forest (RF) model has recently received considerable attention and has been considered an ensemble classification approach with demonstrated superiority and accuracy^28^. However, a major drawback of RF is that it does not work for datasets with insufficient features^29^. Recently, the support vector machine (SVM) model was recognized for its extremely high accuracy compared with that of PLS-DA^30^. Its reputation has been steadily increasing in the biochemical field and has been considered an optimal classifier in many studies because of its extremely high accuracy^31^. The SVM model is advantageous compared with other models because of its capacity to treat high-dimensional and nonlinear data. In particular, an SVM model based on the structural risk minimum (SRM) provides an easy-to-compute solution, is flexible, and shows high generalization capacity^32^. Zhang et al. reported that SVM models built on the reflectance of HSI in the short-wave infrared range (874–1734 nm) could classify the ripeness of strawberries (unripe, mid-ripe, and ripe) at the optimal wavelengths obtained from the loadings of PCA models^33^. Huang et al. demonstrated that the SVM model is a more accurate classifier than the PLS-DA model for classifying mealiness in apples (mealy vs. non-mealy apples)^34^.

In this study, we investigated the potential of HSI processing techniques at different spectral ranges based on near-infrared (NIR) region for classifying napa cabbage quality using nondestructive measurement. The specific objectives were determining the physicochemical and microbiological quality properties of napa cabbage for intercomparison of HSI information, screening the HSI characteristic of 900‒1700 nm spectral ranges for evaluating napa cabbage freshness, and suggesting a novel approach for the classification of healthy and rotten napa cabbage. Consequently, the spectral preprocessing algorithms were verified, and different classification algorithms, such as PLS-DA, SVM, and RF, were used for rapid classification of soft rot symptoms from random napa cabbage spectra.

## Results and discussion

### Values of microbiological and physicochemical properties

#### Evaluation of PCC population in napa cabbage samples

**Figure 1:**
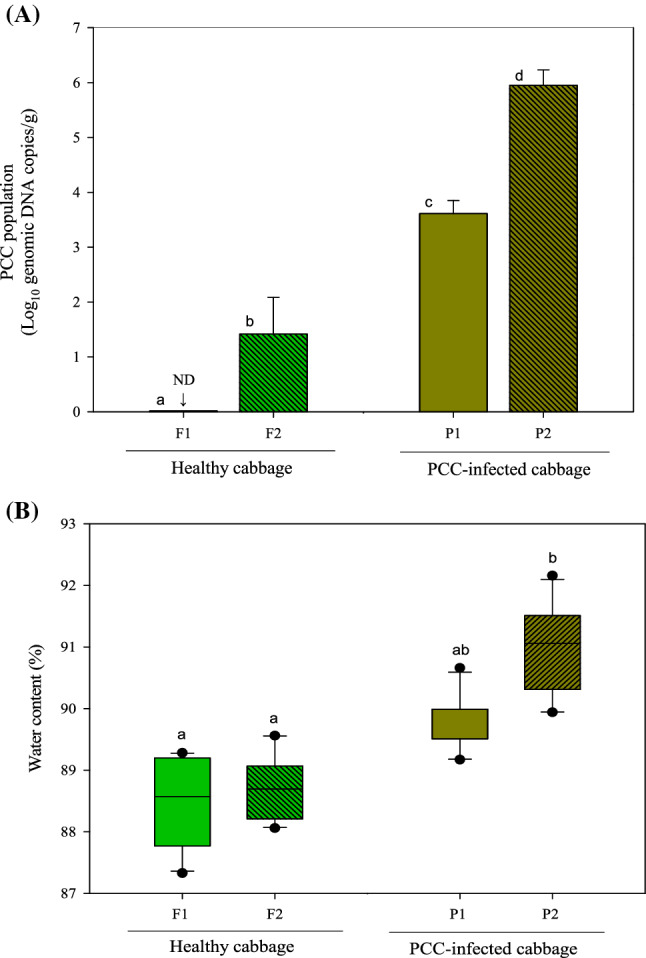
The quantity of *Pectobacterium carotovorum* ssp. *carotovorum* cells recovered from napa cabbage (**A**) and water content of cabbage samples (**B**). Healthy napa cabbage stored at 5 °C (F1), healthy napa cabbage stored at 30 °C (F2), napa cabbage inoculated with PCC and stored at 5 °C (P1), and napa cabbage inoculated with PCC and stored at 30 °C (P2).

After storage, changes in the microbial and physicochemical properties of napa cabbage were analyzed, and the experimental results were used to determine potential soft disease symptoms in the cabbage. The mean titers of PCC in the prepared napa cabbage samples in the F2 (healthy napa cabbage stored at 30 °C), P1 (napa cabbage inoculated with PCC and stored at 5 °C), and P2 (napa cabbage inoculated with PCC and stored at 30 °C) sample groups were 1.41 ± 0.62, 3.61 ± 0.23, and 5.95 ± 0.27 log colony forming unit/g, respectively (Fig. 1A), whereas viable PCC was not detected in the healthy cabbage group (F1). PCC can exist in healthy produce without causing rot^2,35^. It does not grow in contaminated agricultural products unless they are exposed to certain conditions, such as high temperature or external physical forces (e.g., frictional force and shearing force). Thus, although contaminated with PCC, agricultural products will not show symptoms of soft rot disease. The PCC detected in the healthy cabbage group stored at 30 °C likely proliferated during the storage period, but remained in the latent state. Moreover, the discrepancy in the mean titer of PCC between the P1 and P2 groups is presumed to be due to the storage temperature. It has been reported that the optimal growth temperature for *Pectobacterium* spp. is within the range of 20‒34 °C^3,36^. The differential PCC mean values in the four groups of napa cabbage are considered suitable in a reasonable range for separating healthy samples, infected samples, infected but healthy samples, and samples that are healthy but contain PCC cells.

#### Evaluation of water content in napa cabbage samples

Figure 1B presents the descriptive statistics of the water content measured in the four groups of napa cabbage. The highest water content was detected in P2, and it was significantly different from that in other groups; the water content was relatively uniform in both healthy cabbage groups (F1 and F2) and infected cabbage groups (P1 and P2), ranging from 85.4 to 93.8%. The difference in water content of napa cabbage has been attributed to the mean values of the PCC titer—the higher the mean values of the PCC titer, the higher the water content. The variation in water content in various napa cabbage samples could be related to the maceration of cabbage tissues^4,37^.

#### Evaluation of VOCs in napa cabbage samples

**Figure 2:**
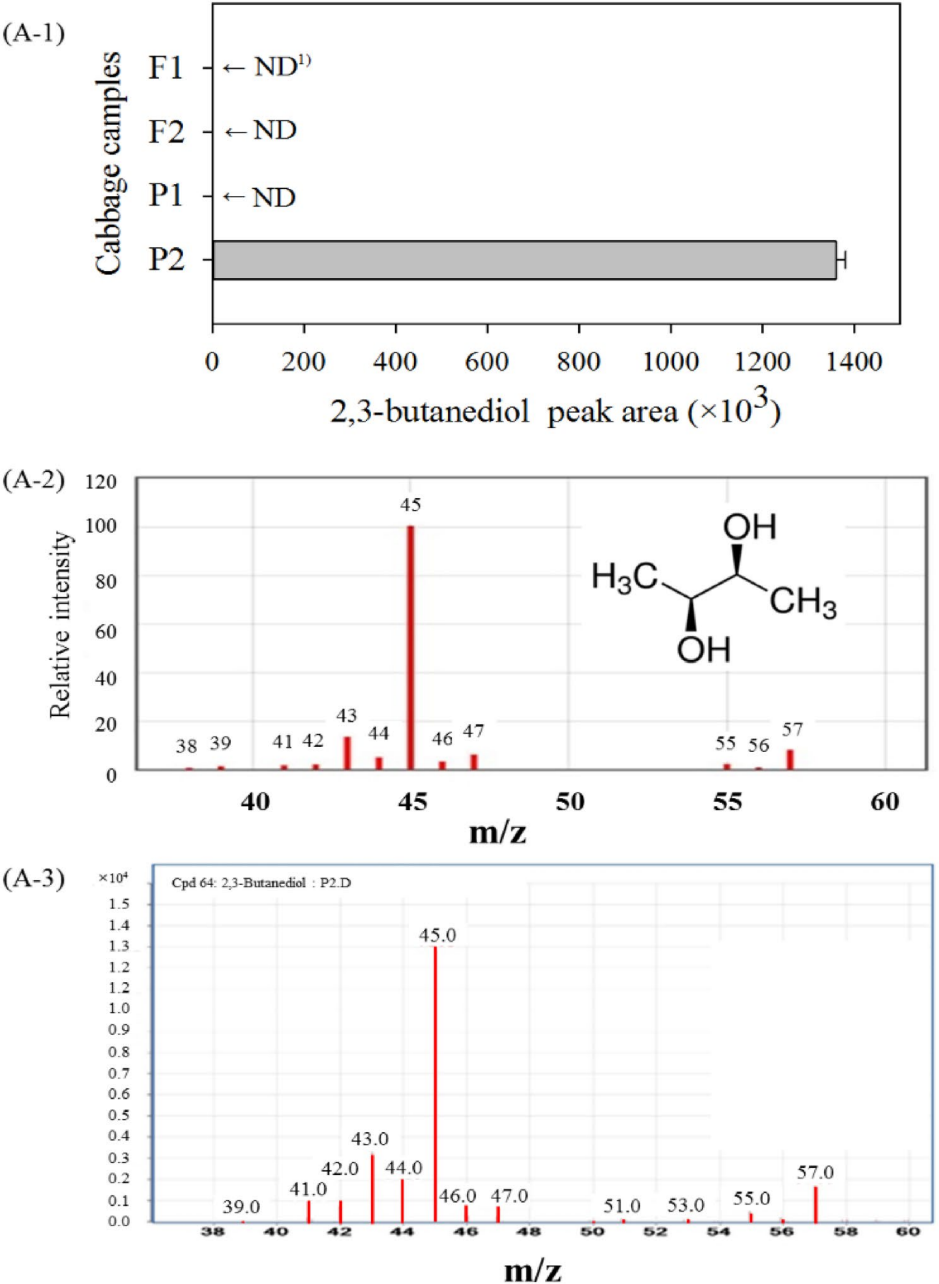
A headspace solid-phase microextraction coupled with gas chromatography–mass spectrometry (HS–SPME–GC–MS) chromatogram. (**A-1**) Volatile compounds in four kinds of napa cabbage samples at different conditions. The area values were obtained from values integrated from area of peaks on Total Ion Chromatogram Total Ion Chromatogram. Healthy napa cabbage stored at 5 °C (F1), healthy napa cabbage stored at 30 °C (F2), napa cabbage inoculated with PCC and stored at 5 °C (P1), and napa cabbage inoculated with PCC and stored 30 °C (P2); (**A-2**) a standard solution containing 2,3-butanediol; (**A-3**) 2,3-Butanediol in napa cabbage samples showing soft rot disease symptom.

For specific detection of VOCs as a marker of soft rot disease, cabbage samples stored for three days at designated temperatures were exposed to the headspace, including the SPME fiber. Table S1 shows the various VOCs emitted by the four types of napa cabbage sample groups and detected by headspace solid-phase microextraction (HS-SPME)–GC–MS analysis. Among cabbage samples with confirmed presence of PCC (F2, P1, and P2), only the P2 group emitted 2,3-butanediol as a volatile marker (Fig. 2). Using HS-SPME combined with GC–MS analysis, Yang et al. demonstrated that 2,3-butanediol is a specific volatile metabolic marker of soft rot disease symptoms in PCC-infected cabbage. The 3-hydroxy-2-butanone pathway is required for *P. carotovorum* pathogenesis, and 2,3-butanediol plays an important role in the production of volatile products^1^. Furthermore, annotation using the KEGG database revealed that butanoate metabolism, which contributes to the rotting scent, is associated with metabolic pathways in *P. carotovorum* subsp. *carotovorum* strain PCC21 (isolated from *B. rapa* ssp. *pekinensis*) (Fig. S1). In the present study, to clearly differentiate the sample group with soft rot disease symptoms, all groups were analyzed using VOC fingerprinting by headspace capillary-gas chromatography-ion mobility spectrometry (HS–GC–IMS). HS–GC–IMS assay for VOC profiling is a highly efficient and automatable analytical technique with acceptable sensitivity (SENS) to VOCs^38^. Based on the representative HS–GC–IMS spectral information of the napa cabbage samples (Fig. S2A1–6,B), 11 target VOC fingerprinting spots were determined to be reliable markers for differentiating soft rot disease symptoms. These VOC fingerprinting spots were investigated in the unsaturated region of the IMS spectra to ensure the validity of the experimental data. Multivariate data were analyzed using unsupervised PCA, which was performed considering Cattell’s scree test, Kaiser’s eigenvalue-one criterion, and Bartlett’s test of sphericity. To compare the experimental groups, multivariate PCA was performed based on normalization using MetaboAnalyst 4.0. According to Tabachnick et al., Kaiser–Meyer–Olkin (KMO) test values above 0.5 are considered acceptable, above 0.9 are excellent, above 0.8 are great, and above 0.7 are good^39^. In this study, the KMO value was above 0.9, considering the interdependence of the components in the PCA. The PCA score plot was constructed to distinguish between the soft rot symptom group (P2) and the non-soft rot symptom groups (F1, F2, and P1) based on the 11 selected VOC fingerprinting spots. The results showed that principal components 1 and 2 explained 98.1% of the variation between the two groups (Fig. S2C).

### Spectral image analysis

**Figure 3:**
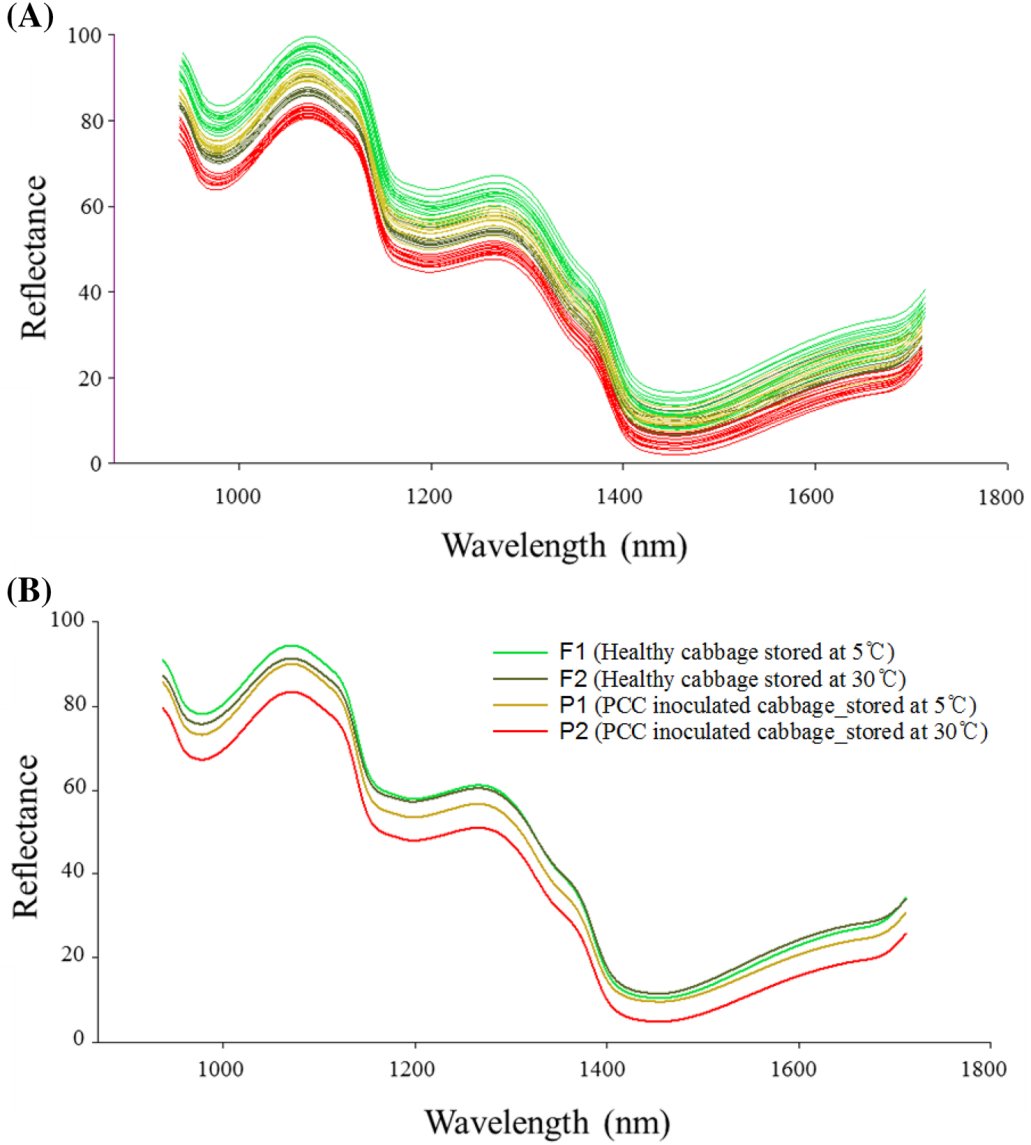
The reflectance spectra of napa cabbages of all samples (**A**) and average spectra for different napa cabbage sample groups (F1, F2, P1, and P2) (**B**).

Changes in the microbial and physicochemical properties of napa cabbage were analyzed to determine whether the cabbage had soft disease symptoms. A total of 197 bands between 938 and 1,711 nm were selected as effective bands owing to obvious noise in the rear and front regions of the spectral data. Figure 3A shows a raw spectrum plot and Fig. 3B a mean raw spectrum plot from the selected region of interest (ROI). Similar spectrum patterns were observed in all napa cabbage samples without any noticeable spectral differences. However, the spectral reflectance of the P2 group was lower than those of the F1, F2, and P1 groups (Fig. 3B) when compared with the mean spectral plot. The napa cabbage samples had absorption peaks at approximately 1180 nm and 1450 nm, which could have been induced by plant stress triggered by various external factors (e.g., bacterial infection, light, or temperature). Particularly, phytopathogenic bacteria can be responsible for changes in relative water content and cellulose^40^. According to Andro et al., PCC produces various enzymatic substances, such as cellulase, extracellular pectinases, and proteases that degrade numerous plant cell wall components^41^. Moreover, cellulase and extracellular pectinases are major virulence factors for the development of soft rot symptoms, which then promote hydration of plant cell tissues^42^. Therefore, it was concluded that the plant disease mechanism of PCC is correlated with the spectral characteristics of napa cabbage samples.

### Classification models based on selected wavelengths

**Figure 4:**
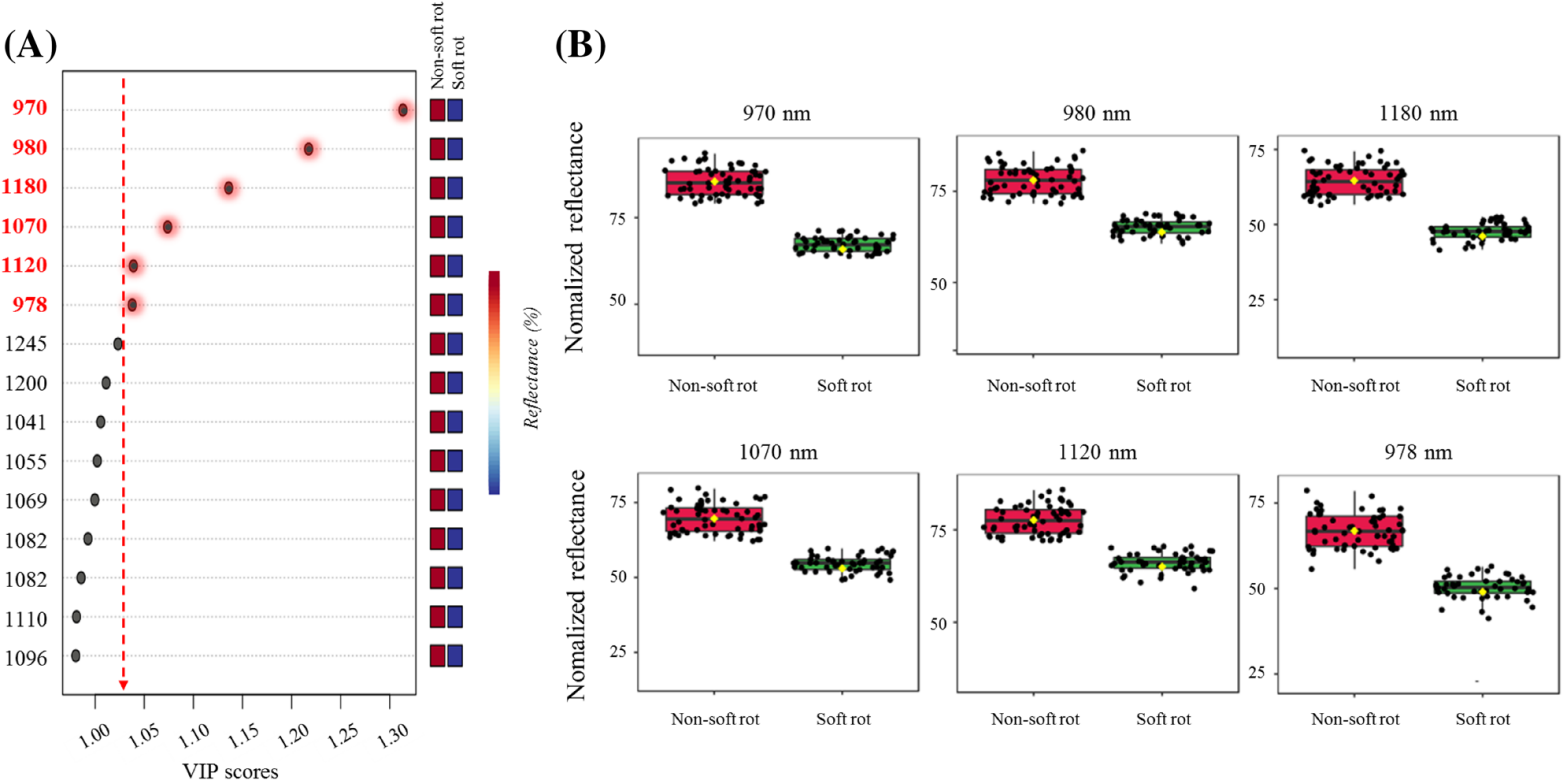
Significant wavelengths by the PLS-DA model, which efficiently classifies soft rot disease infection (**A**), and the comparison of boxplot of the top six significant relatively important wavelengths (p < 0.05) in four groups of napa cabbage samples (**B**).

A PLS-DA classification model was developed to distinguish between the soft rot symptom group and the non-soft rot symptom group of napa cabbage. For this, it was important to prepare a cabbage sample in which each characteristic is clearly defined. Four groups of napa cabbage samples were prepared. According to the microbiological and physicochemical properties, F1, F2, and P1 samples were designated as the non-soft rot disease symptom groups, and P2 samples were designated as the soft rot disease symptom group. The spectra data were enhanced by applying spectra pre- processing to the original spectra based on the Savitzky–Golay’s second derivative (Fig. S3). The Savitzky–Golay’s second derivative is one of the most frequently used techniques for spectral preprocessing to improve the accuracy of calibration models. Only a few optimal wavelengths that convey the most important information representing the entire spectrum were selected to reduce the high dimensionality of the extracted spectral data. We presented the most important wavelengths using the PLS-DA model, which efficiently classifies soft rot disease symptoms, as shown in Fig. 4. Wavelengths in the HSI range were 970, 980, 1,180, 1,070, 1,120, and 978 nm, and they are associated with water absorption, water sensitivity, total chlorophyll, texture, and internal chemical composition^43,44^. Particularly, texture, cellulose, and water sensitivity are key factors for evaluating soft rot symptoms^41,45^. The point just above the VIP score ‘1’ in the SWIR plot was neglected because it fell in the poor SNR region. The number of variables was reduced from 197 to 6 wavelengths in the HSI range on the basis of variable selection for HSI using the VIP scores (Fig. 4). Table 1 presents the confusion matrices of the model with selected wavelengths and the ability of the three algorithms (SVM, PLS-DA, and RF) as classifiers to discriminate soft rot samples from non-soft rot samples. Confusion matrices for the three classifiers indicated that the classification of soft rot napa cabbages was acceptable. The SVM model showed 99% SENS, 96% specificity (SPEC), and 99% ACC for calibration sets and 96% SENS, 88% SPEC, and 95% ACC for validation sets of the napa cabbages (Table 1). The overall cross-validation analysis confirmed that the SENS, SPEC, and ACC were significantly superior in the SVM models than in PLS-DA and RF.

**Table 1:** Confusion matrices for discrimination of napa cabbages inoculated with *Pectobacterium carotovorum* subsp. *carotovorum* (PCC) and healthy napa cabbages based on the pre-processed data in hyperspectral wavelength ranges.

		Confusion matrix (%)				SENS	SPEC	ACC	CV ACC
SVM	Calibration	PV	AV						
				0	1				96%
			0	132	2	99%	96%	99%	
			1	0	45				
	Validation	PV	AV						
				0	1				
			0	43	2	96%	88%	95%	
			1	1	14				
PLS-DA		Confusion matrix (%)				SENS	SPEC	ACC	CV ACC
	Calibration	PV	AV						
				0	1				92%
			0	129	6	96%	88%	96%	
			1	1	44				
	Validation	PV	AV						
				0	1				
			0	41	4	91%	78%	92%	
			1	1	14				
Random forest		Confusion matrix (%)				SENS	SPEC	ACC	CV ACC
	Calibration	PV	AV						
				0	1				89%
			0	129	6	96%	88%	95%	
			1	3	42				
	Validation	PV	AV						
				0	1				
			0	39	6	87%	68%	87%	
			1	2	13				

*SENS* sensitivity, *SPEC* specificity, *ACC* accuracy, *CV* calibration value, *AV* actual values, *PV* predicted values.

## Conclusions

Herein, we report chemometric tools combined with an active sensing system based on HSI spectra (874–1734 nm) for rapid detection and efficient classification of soft rot disease symptoms in napa cabbage. The proposed method successfully classified even PCC-infected cabbage samples that did not emit 2,3-butanediol as a marker volatile substance of soft rot disease. Particularly, although there were no specific symptoms that could be observed with the naked eye, the SVM model accurately distinguished the soft rot disease symptom group. Consequently, the experimental results of this study suggest the potential of HIS using the SVM model, especially under the most effective wavelengths (970, 980, 1180, 1070, 1120, and 978 nm), in separating napa cabbage infected with soft rot from healthy napa cabbages prior to processing. In addition, it was demonstrated that these results can be successfully applied to multispectral images for industrial purposes.

## Methods

### Ethics statement

Our study complied with the relevant institutional, national, and international guidelines and legislation.

### Napa cabbage preparation

**Figure 5:**
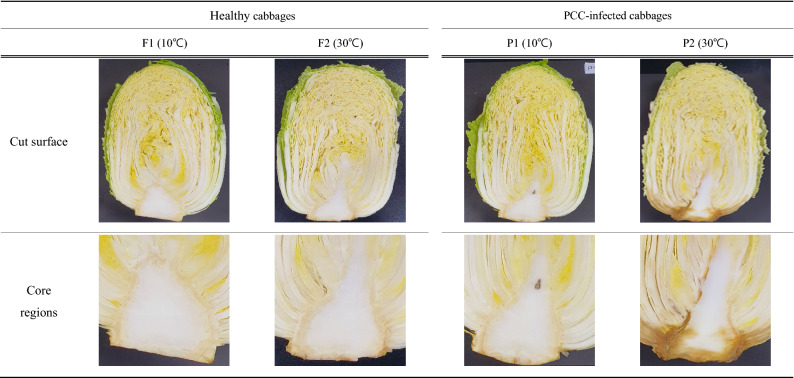
Comparison of *Pectobacterium carotovorum* subsp. *carotovorum* (PCC) bacterial counts and soft rot development between healthy cabbages and PCC-inoculated cabbages under different storage temperatures after 3 days.

The PCC strain KACC 18,645 isolated from napa cabbage was kindly donated by the Rural Development Administration (Deokjin-gu, Jeonju-si, Korea). Healthy napa cabbage was purchased from a local agricultural wholesale market (Gwangju, Korea). The napa cabbages were divided into four groups (30 per group; total of 120 whole cabbages) for conducting the following four treatments: healthy napa cabbage stored at 5 °C (F1), healthy napa cabbage stored at 30 °C (F2), napa cabbage inoculated with PCC and stored at 5 °C (P1), and napa cabbage inoculated with PCC and stored at 30 °C (P2). High temperature (30 °C) was used to simulate the summer ambient conditions. The storage period lasted 3 days. The weight of each napa cabbage was 4000 ± 200 g. For artificial PCC inoculation (P1 and P2), the cut side of the bottom of napa cabbage (core part) was immersed in PCC suspension (approximately 5 log_10_ colony forming unit/mL) for 2 h at 20 °C. The healthy napa cabbages (F1 and F2) were immersed in sterile water for 2 h at 20 °C. Subsequently, each cabbage was cut into two equal sections (total of 60 pieces per treatment) (Fig. 5). In each treatment group (F1, F2, P1, and P2), 45 pieces were used for calibration groups (total of 180 samples; F1: 45 samples, F2: 45 samples, P1: 45 samples, and P2: 45 samples) to develop the classification models and 15 pieces for the validation group (total of 60 samples; F1: 15 samples, F2: 15 samples, P1: 15 samples, and P2: 15 samples) to verify the fitness of the calibration models. For diagnostic analyses of the soft rot disease in cabbage, viable PCC cells were detected using PMA/RT-qPCR assay^46^, water content was determined using the gravimetric method^47^, and extracellular metabolites of the soft rot microorganisms were identified using headspace solid-phase microextraction followed by gas chromatography coupled with mass spectrometry (HS–SPME–GC–MS)^4^.

### Analysis of microbiological and physicochemical properties

#### Enumeration of PCC populations and water content

Each cabbage section was mixed with 30 mL of PBS in a sterile filter stomacher bag (Seward Limited, London, UK) and evenly blended using a stomacher (SHII M; Elmex, Tokyo, Japan) for 60 s. For bacterial genomic DNA extraction, 20 mL of eluate containing PCC bacterial cells was collected and centrifuged at 8000 rpm for 15 min to concentrate the planktonic cells. A Power-Prep quick DNA extraction kit (Kogene-Biotech Co., Seoul, Korea) was used to purify the extracted bacterial genomic DNA according to the manufacturer’s instructions. The suspension samples of the purified DNA were stored at − 70 °C until further use, and 5 µL of each test sample was used directly for one-step real-time reverse transcription PCR (RT-qPCR). For RT-qPCR amplification, a PowerChek *Pectobacterium carotovorum* Real-Time PCR kit (Kogene-Biotech Co.) was used to amplify a DNA template in a 7500 Fast Real-Time PCR System (Applied Biosystems, Foster City, CA, USA), equipped with Applied Biosystems 7500 Fast Real-Time PCR detection system software (version 3.0; Applied Biosystems), according to the manufacturer’s instructions. The standard curve was prepared by illustrating the mean threshold cycle (*Ct*) value (*n* = 3) obtained using the serial dilutions of a standard PCC DNA transcript for RT-qPCR to quantify the PCC bacterial cells in the cabbage sample; a linear plot was obtained, with a coefficient of determination (*R*^2^) > 0.9988 and a slope of − 3.239, reflecting adequate correlation in the tested range between 1.57 and 6.58 log_10_ genomic DNA copies. The water content of each cabbage sample was measured using an infrared moisture analyzer (Model MB 45; Ohaus, Pine Brook, NJ, USA). All experiments were performed in triplicate with ten samples per trial and evaluated for statistical significance. One-way analysis of variance was performed using the SPSS software. Duncan’s multiple range test was used to compare the differences among the mean values.

#### VOC analysis of napa cabbage samples

The VOCs released from the samples by the HS-SPME fiber were analyzed using a gas chromatography system (Agilent 7890A; Agilent Technologies, Santa Clara, CA, USA) coupled with a mass spectrometer (5977 B; Agilent Technologies). HS-SPME was performed using a multipurpose autosampler (MPS2; Gerstel, Mülheim an der Ruhr, Germany). VOC fingerprinting analysis was performed using gas chromatography-ion mobility spectrometry (FlavourSpec®; G.A.S., Dortmund, Germany). VOCs were analyzed by HS-GC-IMS (FlavourSpec®, Gesellschaft für Analytische Sensorsysteme mbH (G.A.S.), Dortmund, Germany) using a 20 cm long × 3 mm ID multicapillary column (MCC) comprising 900 parallel glass capillaries (ID = 40 μm) filled with 20% trifluoropropyl–80% polydimethylpolysiloxane as the stationary phase (film thickness = 0.2 μm; Multichrom Ltd., Novosibirsk, Russia). The injection rate equaled 500 μL, and the carrier flow rate equaled 30 mL s^−1^. For HS–GC–IMS analysis, a finely powdered sample (approximately 100 mg) without any extra pretreatment placed into a 20-mL headspace vial closed with a magnetic cap was incubated at 30 °C for 10 min, and 500 μL of the headspace was automatically injected into the instrument. To avoid cross-contamination, the injector temperature was set up to 80 °C, and the syringe was automatically flushed with a stream of nitrogen for 2 min. Nitrogen was used as a carrier gas (MCC inlet pressure = 2 bar) and was passed through the injector to insert the sample into the GC column, which was heated to 40 °C for timely separation. Molecules were ionized using a tritium source (6.5 keV), and the fragment ions were driven to the drift region using a shutter grid (Bradbury and Nielson design). The 5-cm-long drift tube was operated at a constant voltage of 400 V cm^−1^. The capillary column was held at 45 °C, and the flow rate of drift nitrogen gas was varied as follows: 2 mL min^−1^ for 2 min, 30 mL min^−1^ for 8 min, 100 mL min^−1^ for 10 min, and 150 mL min^−1^ for 5 min. The retention index (RI) of each compound was calculated using n-ketones C4–C9 (Sinopharm Chemical Reagent Beijing Co., Ltd., China) as external references. VOCs were identified by comparing the experimental RI and drift time values with those of the GC–IMS library.

### HSI data acquisition

#### HSI system

A line-scanning type (push-broom scanner) NIR-HSI system (N17E-QE; SPECIM, Spectral Imaging Ltd., Oulu, Finland) was employed to collect the HSI data for napa cabbage samples. The NIR-HSI system used for the analysis was composed of an SWIR camera equipped with an OLES56 camera lens (SWIR-CL-400-N25E; SPECIM) covering the spectral wavelength range of 874–1734 nm with 320 × 256 pixels, a spectral resolution of approximately 12 nm, and a translating scanner. The system consisted of a plate conveyor driven by a stepper motor (Isuzu Optics Corp, Zhubei, Taiwan) and two 150 W tungsten halogen lamps (Fi ber-Lite DC950 Illuminator; Dolan Jenner Industries Inc., Boxborough, MA, USA) fixed symmetrically on both sides of the camera at a 45° angle as the illumination source. The system was placed in a dark room and was controlled using a computer. To scan each sample, each spectral image was acquired for 5 min under controlled ambient temperature (20 °C) in the dark chamber.

#### Image acquisition and correction

The exposure time of the camera, scanning speed of the plate conveyor, and distance between the napa cabbage sample and lens are the main factors influencing the HSI data. Therefore, to collect non-deformable and clear images containing whole napa cabbage samples, three main factors must be accurately set. In this study, the main influencing factors were controlled using LUMO® software (SPECIM). The scanning speed and distance between the lens and samples were set as 22.5 mm/s and 31 cm, respectively. For image correction, both the hyperspectral image acquisition and white/dark reference images were acquired under the same experimental conditions. To remove dark noise and uneven illumination, both hyperspectral image acquisition and white/dark reference images were acquired under the same experimental conditions. A piece of white Teflon (99% reflectance) was used to acquire the white reference image, and the dark reference image was captured by turning off the light source and completely covering the camera lens using an opaque cap. Finally, the corrected images were calculated using the following equation:

$${I}_{C}=\left[ \frac{{I}_{O}-{I}_{D}}{{I}_{W}-{I}_{D}} \right]\times 100,$$

where *I*_*C*_ is the calibrated image, *I*_*O*_ is the original hyperspectral image, *I*_*D*_ is the dark reference image, and *I*_*W*_ is the white reference image. Sample spectral images were extracted using ENVI 4.7 software (Research Systems Inc., Boulder, CO, USA). For hyperspectral images ranging from 938 to 1710, the ROI must be predefined for the extraction of spectral information. In this study, the ROI of each sample was determined to be an orthogonal section of each napa cabbage, from which the spectral data were extracted. Finally, the mean spectral information of each sample was acquired by averaging all pixels of the ROI for additional analysis.

### Model development and performance evaluation

#### Data preprocessing and data analysis

Preprocessing was conducted to improve the accuracy of the classification model and eliminate the influence of irregularities in the spectral data induced by sample texture, light scattering, and random noise. In this study, the Savitzky–Golay’s derivative method for data preprocessing was used to ensure the reliability of the models.

#### Feature selection

Selecting the variable with the highest weight is essential for a quick, simple, and efficient implementation of the PLS-DA model in an image-information-based classification system. Important wavelength selection was obtained by estimating variable importance in projection (VIP) scores of PLS-DA models^27^. Generally, VIP scores < 0.8 represent variables that are less effective, while VIP scores > 1.0 represent highly dominant variables^48^. All chemometric techniques were implemented using the data processing server MetaboAnalyst 4.0^49^.

#### Model development and performance evaluation

Classification models can induce convergence instability because the HSI, as high-dimensional imaging data, presents a high degree of interband correlation and results in data redundancy. To overcome these technical limitations and improve classification stability and implementation of this models in a multispectral imaging system, only optimal wavebands were selected. The combination of the preprocessing method and the developed model achieving the highest accuracy was accepted to be the most suitable for differentiating between napa cabbage with or without soft rot symptoms. The regression coefficient vectors multiplied by the original masked HSI images were employed with the combination of the preprocessing method and model to develop the chemical imaging data. The obtained chemical images were converted into binary images using a threshold value. Resulting chemical images based on pixels denoted "0 value" were classified as non-soft rot symptom group, and those with "1 value" were classified as soft rot symptom group. Binary chemical images were employed to eliminate tiny pixels that caused data misclassification, and the binary chemical images were further clarified. Preprocessing was conducted to increase the accuracy of the classification model and eliminate the bias due to irregularities in the spectral data induced by sample texture, light scattering, and random noise. In this study, prior to the application of classification models, the Savitzky–Golay’s derivative method was used as a preliminary operation to exclude outliers. Three multivariate models, SVM with linear kernel, PLS-DA, and RF, were applied to classify the soft rot and non-soft rot symptom groups of napa cabbage using MetaboAnalyst 4.0. Multivariable analysis procedures have been recognized as standard approaches and are widely used in the analysis of HIS images. Hyperspectral data analysis aims to develop a classification or a predictive model. In this study, pre-processed hyperspectral data from individual methods were employed for developing a classification model between the soft rot and the non-soft rot symptom groups of napa cabbage.

### Multivariate data analysis

SVM, PLS-DA, and FR were used as the classifiers. According to Barker and Rayens, the PLS-DA model utilizes the interaction between sample characteristics and spectral intensities by maximizing the covariance between variables^50^. The performance of the developed SVM, PLS-DA, and FR models was investigated based on SENS, SPEC, and ACC, representing the true positive rates, true negative rates, and overall correctly classified samples, respectively.

$$Specificity= \frac{True negatives}{(True negarives+False positives)},$$

$$Sensitivity= \frac{True positives}{(True positives+False negatives)},$$

$$Accuracy= \frac{True positives+ True negatives}{(True positives+True negatives+False positives+False negatives)},$$

where *n* is the number of samples. The accuracy of the SVM, PLS-DA, and FR model can be improved by choosing the optimal number of latent variables on the basis of the minimum root mean square error of cross-validation.

## Supplementary Information

Supplementary Information.

## Data Availability

The datasets used and analyzed during the present study are available from the corresponding author on reasonable request. The data related to the major pathway maps of differential metabolites by *Pectobacterium carotovorum* subsp. *carotovorum* is available at https://www.kegg.jp/pathway/map00650.
