# Supplementary material for: Nondestructive classification of soft rot disease in napa cabbage using hyperspectral imaging analysis
Source: Sci Rep. 2022 Aug 29;12:14707. doi: 10.1038/s41598-022-19169-6 (PMC9424267; doi:10.1038/s41598-022-19169-6)
Supplement: Supplementary file 1 — Supplementary Information. [file 41598_2022_19169_MOESM1_ESM.docx]

**Supplementary Materials**

**Nondestructive classification of soft rot disease in napa cabbage using hyperspectral imaging analysis**

Hyeyeon Song^1^, So-Ra Yoon^1^, Yun-Mi Dang^1^, Ji-Su Yang^1^, In Min Hwang^1^, Ji-Hyoung Ha^1^*

^1^Hygienic Safety and Distribution Research Group, World Institute of Kimchi, Gwangju, 61755, Republic of Korea

**NUMBER OF TABLES: 1**

**NUMBER OF FIGURES: 3**

* Corresponding author:

Ji-Hyoung Ha

Hygienic Safety and Analysis Center World Institute of Kimchi

86 Kimchi-ro, Nam-gu, Gwangju 61755, Republic of Korea

Tel: 82-62-610-1845

Fax: 82-62-610-1810

**List of Contents**

**Tables:**

**Table S1**. Volatile compounds in napa cabbage samples at different storage temperatures.

.

**Figures:**

**Figure S1.** The major pathway maps of differential metabolites in butanoate metabolism by *Pectobacterium carotovorum* subsp*. carotovorum*.

**Figure S2.** Topographic plots of VOC fingerprints (A) and gallery plots (B) of selected VOCs for napa cabbage samples by HS-GC-IMS assay. (C) PCA score plot showing the classification for napa cabbages.

**Figure S3.** Savitzky-Golay 2nd derivative pre-processed spectra of napa cabbages.

**Table S1. Volatile compounds in napa cabbage samples at different storage temperatures.**

| No. | Volatile compounds | Formula | ^1)^Peak area (×10^3^) | | | | |
| --- | --- | --- | --- | --- | --- | --- | --- |
|  |  |  | BK | F1 | F2 | P1 | P2 |
| 1 | 1-Penten-3-one | C5H8O | - | - | - | 112 | 248 |
| 2 | 3-Ethyl-1,5-octadiene | C10H18 | - | - | - | - | 38 |
| 3 | 3-Methyl-1-heptene | C8H16 | - | 77 | 195 | - | - |
| 4 | 1-Chloroheptane | C7H15Cl | - | - | - | 78 | 159 |
| 5 | 2,3-Pentanedione | C5H8O2 | - | - | 138 | - | 85 |
| 6 | Dimethyl disulfide | C2H6S2 | - | 257 | 165 | 336 | 943 |
| 7 | Hexanal | C6H12O | - | 95 | 375 | 66 | 338 |
| 8 | 4-Ethyl-1-hexyn-3-ol | C8H14O | - | - | - | - | 47 |
| 9 | Dihydropyran | C5H8O | - | - | - | - | 90 |
| 10 | *(E)*-2-pentenal | C5H8O | - | - | 51 | 27 | 68 |
| 11 | Di-*tert*-butyl dicarbonate | C10H18O5 | - | - | 130 | 67 | - |
| 12 | 1-Penten-3-ol | C5H10O | - | - | - | 59 | 94 |
| 13 | 2-Methyl-1-methylsulfanylbut-2-ene | C6H12S | - | - | - | - | 14 |
| 14 | 2-Pyridinecarboxylic acid | C6H5NO2 | - | - | - | - | 14 |
| 15 | Methanesulfonyl chloride | CH3ClO2S | - | 1011 | 870 | 160 | 186 |
| 16 | 4-Methyl-2-heptanone | C8H16O | - | - | - | - | 19 |
| 17 | *N*-(3-methylphenyl)-2-acetoxy-acetamide | C11H13NO3 | - | - | 17 | - | - |
| 18 | Isopentyl alcohol | C5H12O | - | - | - | 188 | 312 |
| 19 | *(E)*-2-Hexenal | C6H10O | - | 100 | 190 | 114 | 91 |
| 20 | 2-Pentylfuran | C9H14O | - | 133 | 147 | 64 | 602 |
| 21 | 2,3-Epoxy-4,4-dimethylpentane | C7H14O | - | - | - | - | 19 |
| 22 | Butyl isothiocyanate | C5H9NS | - | 31 | 84 | 64 | 161 |
| 23 | 3-Pentenenitrile | C5H7N | - | 375 | - | - | - |
| 24 | Methyl thiocyanate | C2H3NS | - | - | 387 | 1552 | 3409 |
| 25 | Acetoin | C4H8O2 | - | - | - | - | 177 |
| 26 | *(E)*-2-Pentenylfuran | C9H12O | - | 20 | 16 | 20 | 12 |
| 27 | 4-Pentenol | C5H10O | - | - | 21 | 19 | 20 |
| 28 | Cyclopentanol | C5H10O | - | - | - | - | 16 |
| 29 | Vinyl hexanoate | C8H14O2 | - | - | 126 | 69 | 46 |
| 30 | *(E)*-2-Heptenal | C7H12O | - | 26 | 29 | 36 | 166 |
| 31 | *(Z)*-2-Heptenal | C7H12O | - | - | 108 | 61 | - |
| 32 | *(Z)*-2-Pentenol | C5H10O | - | - | - | 137 | 155 |
| 33 | 1-(2-Aminophenyl)-3-*tert*-butylurea | C11H17N3O | - | - | - | 20 | 31 |
| 34 | 6-Octen-2-one | C8H14O | - | - | 12 | - | - |
| 35 | 5-Hexenenitrile | C6H9N | - | 2262 | 3970 | 3081 | 5083 |
| 36 | 1-Hexanol | C6H14O | - | - | - | - | 136 |
| 37 | 5-Methylhexanenitrile | C7H13N | - | 40 | 119 | 63 | 137 |
| 38 | Isoamyl cyanide | C6H11N | - | 11 | 23 | 24 | 18 |
| 39 | Dimethyl trisulfide | C2H6S3 | - | 564 | - | 483 | 430 |
| 40 | *(Z)*-3-Hexen-1-ol | C6H12O | - | 61 | 193 | 30 | 144 |
| 41 | Nonanal | C9H18O | - | 39 | 18 | 17 | 17 |
| 42 | Heptanonitrile | C7H13N | - | 25 | 40 | 46 | 48 |
| 43 | 3-Cyclohexylpropyl alcohol | C9H18O | - | 11 | 38 | 26 | - |
| 44 | 3-Octen-2-one | C8H14O | - | - | 22 | 16 | - |
| 45 | 6-Methoxy 2-hexanone | C7H14O2 | - | - | - | - | 13 |
| 46 | 1,3-Di-*tert*-butylbenzene | C14H22 | - | 141 | 123 | 55 | 142 |
| 47 | *(E)*-2-Octenal | C8H14O | - | 13 | 29 | 34 | 31 |
| 48 | *p*-Cymene | C10H14 | - | 19 | - | - | - |
| 49 | Isodurene | C10H14 | - | - | 18 | - | - |
| 50 | 1-Octen-3-ol | C8H16O | - | 12 | 47 | 25 | 39 |
| 51 | 1-Acetyl-2-methylazetidine | C6H11NO | - | - | - | - | 36 |
| 52 | 3-Butenyl isothiocyanate | C5H7NS | - | 2287 | - | 5377 | 314 |
| 53 | Methoxyacetic acid, 10-undecenyl ester | C14H26O3 | - | - | - | - | 50 |
| 54 | Nonanenitrile | C9H17N | - | 29 | 112 | 66 | 18 |
| 55 | 1-Ethyl-2-trifluoroacetoxycyclohexane | C10H15F3O2 | - | - | - | - | 35 |
| 56 | 6-Heptenenitrile | C7H11N | - | - | - | - | 12 |
| 57 | 4,4-Dimethyl-5-oxopentanenitrile | C7H11NO | - | - | 33 | 27 | - |
| 58 | 2,4-Heptadienal | C7H10O | - | 791 | 1218 | 1703 | 379 |
| 59 | Decanal | C10H20O | - | - | - | - | 15 |
| 60 | 3-Amino-4-anilinobenzonitrile | C13H11N3 | 11 | - | - | - | - |
| 61 | *α*-Bergamotene | C15H24 | - | - | - | - | 67 |
| 62 | 3-Nonen-2-one | C9H16O | - | - | - | - | 31 |
| 63 | Benzaldehyde | C7H6O | - | 657 | 1016 | 890 | 338 |
| 64 | 4-(2-Fluorobenzylidene)-2-phenyl-1,3-oxazol-5(4H)-one | C16H10FNO2 | - | - | - | 47 | - |
| 65 | 2,3-Dimethylfumaric acid | C6H8O4 | - | - | 94 | 412 | - |
| 66 | 4-Ethyl-5-methylthiazole | C6H9NS | - | 3126 | - | 13647 | 753 |
| 67 | 4-Methylpentyl isothiocyanate | C7H13NS | - | 18 | - | 65 | - |
| 68 | 1-Octanol | C8H18O | - | - | 11 | - | 14 |
| 69 | 3,5-Octadien-2-one | C8H12O | - | 398 | 127 | 58 | 49 |
| 70 | *(E,E)*-3,5-Octadien-2-one | C8H12O | - | 431 | 636 | 931 | 34 |
| 71 | *(S,S)*-2,3-Butanediol | C4H10O2 | - | - | - | - | 1360 |
| 72 | 3,3,6-Trimethyl-1,5-heptadiene | C10H18 | - | - | - | 15 | - |
| 73 | Hexyl isothiocyanate | C7H13NS | - | - | - | 27 | - |
| 74 | 6-Methyltridecane | C14H30 | - | 39 | - | - | - |
| 75 | Hexadecane | C16H34 | - | 36 | - | - | - |
| 76 | 1,6-Dimethyl-4-propan-hexahydronaphthalene | C15H24 | - | - | - | - | 50 |
| 77 | (1,1-Dimethyldecyl)benzene | C18H30 | - | - | - | - | 30 |
| 78 | *Z,Z*-2,6-Dimethyl-3,5,7-octatriene-2-ol | C10H16O | - | - | - | - | 12 |
| 79 | 9-Hydroxy-2-nonanone | C9H18O2 | - | - | - | - | 13 |
| 80 | Triepoxy decane | C10H16O3 | - | - | - | - | 10 |
| 81 | Benzyl carbazate | C8H10N2O2 | - | 127 | - | - | 16 |
| 82 | Methyl *N*-hydroxybenzenecarboximidate | C8H9NO2 | - | 2980 | 1976 | 2346 | 3463 |
| 83 | α-Curcumene | C15H22 | - | - | - | - | 38 |
| 84 | Methyl salicylate | C8H8O3 | - | 1057 | 924 | 1109 | - |
| 85 | 3-(2-Phenylethyl)benzonitrile | C15H13N | 114 | 70 | 94 | 105 | - |
| 86 | 3,4-Bis(methoxycarbonyl)benzoic acid | C11H10O6 | - | 148 | 138 | 170 | 62 |
| 87 | Phenylethyl alcohol | C8H10O | - | 282 | 540 | 195 | 237 |
| 88 | Benzyl nitrile | C8H7N | - | - | - | 21 | 10 |
| 89 | 5-Methylthiopentanonitrile | C6H11NS | - | 71 | - | 255 | - |
| 90 | Methyl 5-chloropent-3-enoate | C6H9ClO2 | - | - | 380 | - | 206 |
| 91 | 4,5-Dimethylthiazole | C5H7NS | - | 633 | 440 | 654 | 202 |
| 92 | Isoamyl phenylacetate | C13H18O2 | - | - | - | - | 17 |
| 93 | 1-Chloro-2-methylbutane | C5H11Cl | - | 85 | - | 109 | 98 |
| 94 | Hexadecanal | C16H32O | - | - | - | - | 17 |
| 95 | Benzenepropanenitrile | C9H9N | - | 31099 | 28852 | 49760 | 3102 |
| 96 | Tetrahydro-pyrrolo[1,2-c]oxazole-3-thione | C6H9NOS | - | 706 | 170 | 2560 | 177 |
| 97 | 6-Methylindole | C9H9N | - | - | - | - | 20 |
| 98 | 4,4-Ethylenedioxy-pentanenitrile | C7H11NO2 | - | - | - | - | 434 |
| 99 | Cyclopenta[c]thiophene | C7H12S | - | 1182 | 970 | 1267 | - |
| 100 | Methyl palmitate | C17H34O2 | - | - | - | - | 42 |
| 101 | 2-Phenylethyl isothiocyanate | C9H9NS | - | 28957 | 262 | 31894 | - |
| 102 | D-Galactono-γ-lactone | C6H10O6 | - | 23 | - | - | - |
| 103 | 4,4,6-Trimethyltetrahydro-1,3-thiazine-2-thione | C7H13NS2 | - | - | - | 476 | - |
| Data are shown as the mean (n=3). | |  |  |  |  |  |  |
| - : LOD, Limit of detection.  ^1)^The area values were obtained from values integrated from area of peaks on TIC. | | | | | | | |

Figure S1.


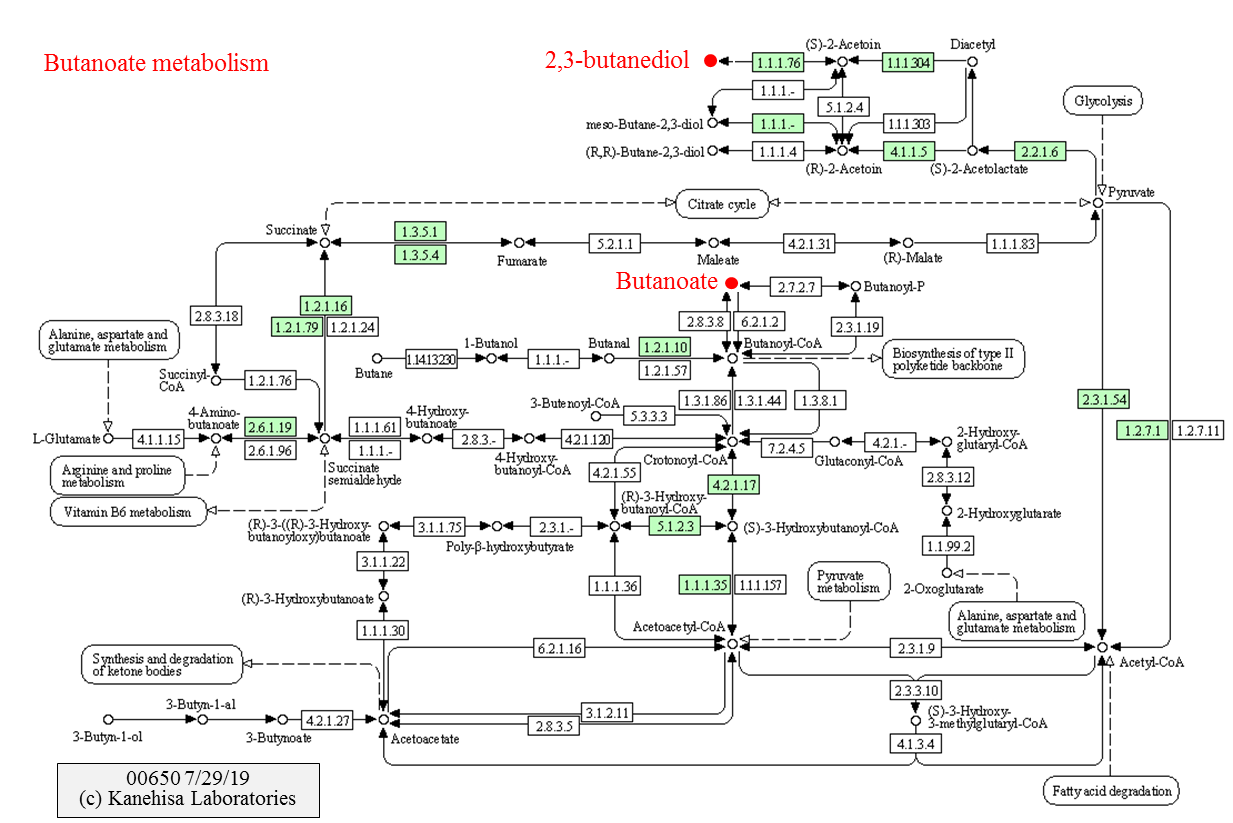


Figure S2.


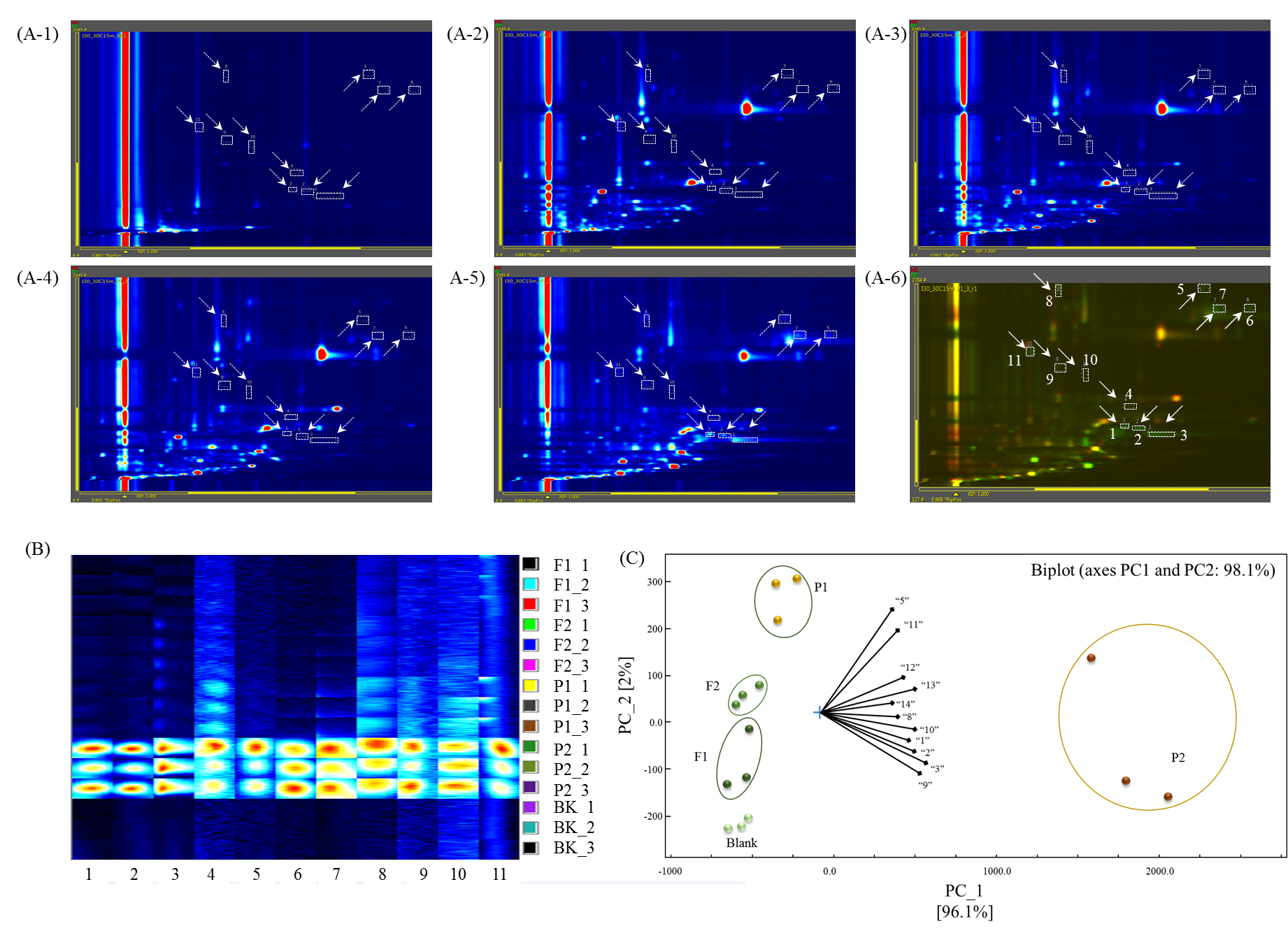


Figure S3.
